# Multidisciplinary integrative care versus chiropractic care for low back pain: a randomized clinical trial

**DOI:** 10.1186/s12998-022-00419-3

**Published:** 2022-03-01

**Authors:** Gert Bronfort, Michele Maiers, Craig Schulz, Brent Leininger, Kristine Westrom, Greg Angstman, Roni Evans

**Affiliations:** 1 University of Minnesota, Mayo Building C504, 420 Delaware Street SE, Minneapolis, MN 55455 USA; 2 Northwestern Health Sciences University, 2501 W. 84th Street, Bloomington, MN 55431 USA; 3 St. Elizabeth’s Medical Center-Wabasha, 1000 1st Dr NW, Austin, MN USA

**Keywords:** Back pain, Multidisciplinary, Integrative medicine, Chiropractic, Clinical trial

## Abstract

**Background:**

Low back pain (LBP) is influenced by interrelated biological, psychological, and social factors, however current back pain management is largely dominated by one-size fits all unimodal treatments. Team based models with multiple provider types from complementary professional disciplines is one way of integrating therapies to address patients’ needs more comprehensively.

**Methods:**

This parallel group randomized clinical trial conducted from May 2007 to August 2010 aimed to evaluate the relative clinical effectiveness of 12 weeks of monodisciplinary chiropractic care (CC), versus multidisciplinary integrative care (IC), for adults with sub-acute and chronic LBP. The primary outcome was pain intensity and secondary outcomes were disability, improvement, medication use, quality of life, satisfaction, frequency of symptoms, missed work or reduced activities days, fear avoidance beliefs, self-efficacy, pain coping strategies and kinesiophobia measured at baseline and 4, 12, 26 and 52 weeks. Linear mixed models were used to analyze outcomes.

**Results:**

201 participants were enrolled. The largest reductions in pain intensity occurred at the end of treatment and were 43% for CC and 47% for IC. The primary analysis found IC to be significantly superior to CC over the 1-year period (*P* = 0.02). The long-term profile for pain intensity which included data from weeks 4 through 52, showed a significant advantage of 0.5 for IC over CC (95% CI 0.1 to 0.9; *P* = 0.02; 0 to 10 scale). The short-term profile (weeks 4 to 12) favored IC by 0.4, but was not statistically significant (95% CI − 0.02 to 0.9; *P* = 0.06). There was also a significant advantage over the long term for IC in some secondary measures (disability, improvement, satisfaction and low back symptom frequency), but not for others (medication use, quality of life, leg symptom frequency, fear avoidance beliefs, self-efficacy, active pain coping, and kinesiophobia). Importantly, no serious adverse events resulted from either of the interventions.

**Conclusions:**

Participants in the IC group tended to have better outcomes than the CC group, however the magnitude of the group differences was relatively small. Given the resources required to successfully implement multidisciplinary integrative care teams, they may not be worthwhile, compared to monodisciplinary approaches like chiropractic care, for treating LBP.

*Trial registration* NCT00567333.

## Background

Low back pain (LBP) is one of the most prevalent and disabling chronic health conditions. An estimated 40–80% of adults experience low back pain (LBP) at some point in their lives [1, 2]. Further, LBP related disability continues to increase, making it a leading cause of disability globally [3]. Approximately 20% of acute cases become chronic [4], and it is these individuals that bear a disproportionate share of LBP associated burden [5]. Importantly, LBP is one of the stronger risk factors for chronic opioid use [6].

While the ‘biopsychosocial model’ for LBP has been promoted for decades, it is still incompletely and inadequately applied in both research and clinical practice [7–11]. Indeed, the majority of back pain cases remain poorly treated with a heavy emphasis on symptom management [11] using a ‘one size fits all’ approach that fails to address sufferers’ unique needs [7, 11–13]. This has resulted in the persistent use of marginally effective and potentially harmful unimodal therapies (injections, drug therapies, etc.) with a primarily physical focus. Further, current back pain management practices often contradict clinical guideline recommendations by failing to offer treatment options with scientific support, including complementary approaches [13–16]. This includes spinal manipulation, exercise, acupuncture, cognitive behavioral therapy, self-care strategies, and others [15, 17, 18].

Integrating complementary modalities with conventional approaches has shown promise for LBP in previous studies [19]. Team based models of care with multiple provider types from complementary professional disciplines has been one way of integrating different therapies to more comprehensively address individual patients’ needs [11, 20]. Such approaches combine a range of viable treatment options to synergistically address multidimensional causes of pain, with the goal of exceeding the therapeutic effect of any one therapy alone [21, 22]. A previous manuscript by our group described one approach for a team based model of care including acupuncturists, chiropractors, psychologists, exercise therapists, massage therapists, primary care physicians with case managers coordinating care [23].

The purpose of this manuscript is to report the primary and secondary clinical outcomes of a randomized trial of monodisciplinary chiropractic care (CC), versus multidisciplinary integrative care (IC) for sub-acute and chronic LBP.

## Methods

This was a parallel group randomized clinical trial funded by the US Department of Health and Human Services. It was conducted from May 2007 to October 2009 with follow-up data collection through August 2010. Institutional Review Boards at participating institutions (Northwestern Health Sciences University Study #1-32-10-06 and Minneapolis Medical Research Foundation Study # 07-2785) approved the study protocol which has been described elsewhere [24]. The study was monitored by a Data Safety and Monitoring Board. Written consent was provided by all participants.

### Settings and participants

The study was conducted at Northwestern Health Sciences University (Minneapolis, Minnesota). Participants were recruited from the surrounding Minneapolis/St. Paul metropolitan area primarily through targeted postcard mailings, brochures at community events, and advertisements in online local newspapers.

### Inclusion criteria

Inclusion criteria were 18 years of age or older, LBP categories 1, 2, 3, or 4 according to the Quebec Task Force classification system (individuals with back pain, stiffness, or tenderness with or without leg pain or neurological signs) [25]; current episode of LBP 6 weeks or longer in duration; and LBP rating of ≥ 3 on a 0–10 scale during the previous week.

### Exclusion criteria

Individuals were excluded if they had contraindications to study treatments (i.e. active inflammatory disease of the spine, blood clotting disorders, or severe osteoporosis) or who were pregnant or nursing, had current or pending spine-related litigation, a history of multiple lumbar surgeries, or progressive neurological deficits.

### Randomization

The study statistician utilized a computer-generated randomization scheme and applied a 1:1 allocation ratio with randomly permuted block sizes that was concealed from the study team. As individuals became eligible, sequentially numbered sealed, opaque envelopes were drawn in consecutive order and opened in the presence of the study participant by the study team.

### Interventions

All participants in the study received 12 weeks of either monodisciplinary chiropractic care (CC) or multidisciplinary team-based integrative care (IC). CC was delivered by a team of chiropractors allowed to utilize any non-proprietary treatment under their scope of practice not shown to be ineffective or harmful including manual spinal manipulation (i.e., high velocity, low amplitude thrust techniques, with or without the assistance of a drop table) and mobilization (i.e., low velocity, low amplitude thrust techniques, with or without the assistance of a flexion-distraction table). Chiropractors also used hot and cold packs, soft tissue massage, teach and supervise exercise, and administer exercise and self-care education materials at their discretion. IC was delivered by a team of six different provider types: acupuncturists, chiropractors, psychologists, exercise therapists, massage therapists, and primary care physicians, with case managers coordinating care delivery. Interventions included acupuncture and Oriental medicine (AOM), spinal manipulation or mobilization (SMT), cognitive behavioral therapy (CBT), exercise therapy (ET), massage therapy (MT), medication (Med), and self-care education (SCE), provided either alone or in combination and delivered by their respective profession. Participants were asked not to seek any additional treatment for their back pain during the intervention period. Standardized forms were used to document the details of treatment, as well as adverse events. It was not possible to blind patients or providers to treatment due to the nature of the study interventions. Patients in both groups received individualized care developed by clinical care teams unique to each intervention arm. Care team training was conducted to develop and support group dynamics and shared clinical decision making. A clinical care pathway, designed to standardize the process of developing recommendations, guided team-based practitioner in both intervention arms. Evidence based treatment plans were based on patient biopsychosocial profiles derived from the history and clinical examination, as well as baseline patient rated outcomes. The pathway has been fully described elsewhere [23]. Case managers facilitated patient care team meetings, held weekly for each intervention group, to discuss enrolled participants and achieve treatment plan recommendation consensus. Participants in both intervention groups were presented individualized treatment plan options generated by the patient care teams, from which they could choose based on their preferences.

To assess response to treatment during the intervention phase, patients completed a Patient Self-Assessment Form (PSAF) at each of their visits which was adapted from the Measure Yourself Medical Outcome Profile [26]. Patients chose a symptom and an activity most affected by their LBP, and then rated it on a 0–10 scale. Treating providers monitored patient progress by assessing patients’ PSAF in relation to benchmarks for improvement generated from previous studies [23]. When benchmarks for improvement were not met, providers brought the case back to their respective care team for review and potential alteration of the treatment plan.

Table 1 describes the specific details of the treatments using the Template for Intervention Description and Replication (TIDieR) [27].

**Table 1:** Description of interventions using the Template for Intervention Description and Replication (TIDieR) [27]

1. Brief name	Monodisciplinary Chiropractic Care (CC) [24]	Multidisciplinary Integrative Care (IC) [24]
2.Why	Rationale: Chiropractors commonly treat LBP patients with evidence-based modalities found to be effective for LBP	Rationale: Given the biopsychosocial nature of LBP, integrating multiple types of evidence-based modalities may exceed the therapeutic effect of any one modality alone; one approach is multidisciplinary integrative care
3. What Materials	Patients: handouts with pictures and descriptions of exercises and self-care postures Providers: manuals of operations, standardized treatment notes	
4. What Procedures	Manual spinal manipulation (i.e., high velocity, low amplitude thrust techniques, with or without the assistance of a drop table) Manual mobilization (i.e., low velocity, low amplitude thrust techniques, with or without the assistance of a flexion-distraction table) Spinal mobility, strength/endurance, and stabilization exercises Adjunct therapies common to clinical practice (i.e. hot and cold packs, soft tissue massage)	Traditional Chinese Medicine (i.e. acupuncture, liquid moxa with a heat lamp, Tui Na, and cupping) Chiropractic care (including spinal manipulation, manual mobilization, adjunct therapies as described in CC group) Cognitive behavioral therapy (i.e. operant and respondent cognitive approaches including environmental restructuring) Rehabilitative exercise (i.e. spinal mobility, strength/endurance and stabilization exercises) Therapeutic massage (i.e. neuromuscular therapy, myofascial techniques, trigger point therapy, and classic western style Swedish massage) Medication (i.e. non-steroidal anti-inflammatory drugs (NSAIDS), analgesics, and/or muscle relaxants) Self-care education (i.e. spine posture awareness for activities of daily living specific to their abilities, such as lifting, pushing and pulling, sitting and getting out of bed)
5. Who	3 licensed chiropractors; met weekly as a team Training included review of evidence for specific modalities; collaborative evidence-based decision making	13 licensed or certified practitioners (3 Traditional Chinese Medicine, 2 chiropractors, 3 massage therapists, 2 psychologists, 1 allopathic physician, and 2 exercise therapists); met weekly as team Study related training included orientation to different treatments and practices (theoretical mechanisms, modalities); review of evidence for specific modalities; collaborative evidence-based decision making
6. How	1:1 visits; in person	
7. Where	Research clinic	
8. When, how much	12 weeks intervention period; number of visits based on individual patient needs; typical visit duration 15–30 min	12 weeks intervention period; number of visits based on individual patient needs; typical visit duration varied by treatment type: Cognitive Behavioral Therapy, Massage Therapy (60 min); Traditional Chinese Medicine, Exercise and Self-Care Education (40–60 min); Chiropractic Care (15–30 min); Medication- 15–30 min
9. Tailoring	Treatment plan options based on care team’s evaluation of the patient profile generated from baseline health history, physical examination findings, and patient rated outcomes measures Treatment plans presented by case manager, and selected by study participant Decision regarding number and frequency of treatment visits based on patient response to treatment (i.e. self-selected symptom and activity ratings) using a Patient Self-Assessment Form	
10. Modifications	None	
11. Planned Fidelity Assessment	Routine monitoring of standardized treatment notes by research staff Patient self-report of out-of-scope care during intervention phase	
12. Actual Fidelity Assessment	3 patients sought additional care during intervention phase	1 patient sought additional care during intervention phase

### Outcomes measures

Participant demographic and clinical characteristics were collected during the baseline visits through self-report questionnaires and a health history and physical examination. Self-reported outcomes were collected at 2 baseline visits (7–14 days apart) and at 4, 8, 12, 26, and 52 weeks after enrollment using questionnaires administered independent of staff or clinician influence.

#### Primary outcome measure

The primary outcome measure was typical level of back pain over the previous week, using a numerical rating scale (0 = no pain, 10 = the worst pain possible) [28–31].

#### Secondary outcome measures

Secondary outcomes included:

- Back disability measured with the 23-item Roland Morris Disability Questionnaire (converted to a 0 to 100 scale) [32, 33]
- Global improvement (1 = no symptoms, 100% improvement; 9 = as bad as it could be, 100% worse) [34, 35]
- Days with medication use for back pain in the past week [36]
- Quality of life measured with the EuroQol EQ5D-3L (− 0.109 to 1) [37]
- Satisfaction with care (1 = completely satisfied, couldn’t be better; 7 = completely dissatisfied, couldn’t be worse) [38, 39]
- Frequency of low back or leg symptoms (0 = none of the time; 5 = all of the time) [32, 40]
- Number of days in the past month with missed work or reduced activities due to back pain [41]
- Work (0 to 42) and physical activity (0 to 24) subscales of the fear avoidance beliefs questionnaire [42]
- Pain self-efficacy (0 to 60) [43]
- Pain coping strategies measured with the Vanderbilt Pain Management Inventory short form (active strategies subscale 5 to 25; passive strategies subscale 6 to 30) [44, 45]
- Kinesiophobia measured with the Tampa Scale for Kinesiophobia (17 to 68) [46].

### Power calculation and sample size

In a previous chronic LBP trial of exercise and spinal manipulation conducted by our team, group differences in pain of up to 8 percentage points after 3 months of treatment were observed. Informed by these results, the scientific literature at the time, and consensus of study investigators and clinicians regarding clinical importance, we were interested in detecting an 8-percentage point between group difference in pain after 12 and 52 weeks of treatment. Based on an α of 0.05 and 80% power, 85 participants per group were required. An allowance of 15% drop-out rate resulted in a total sample size of 200 participants.

### Statistical analysis

We used an intention-to-treat approach, analyzing all observed data from participants according to their allocated treatment assignment. Data were prepared for analysis by a data manager masked to group status; analyses were performed in SAS, version 9.1.

The primary and most secondary outcomes were analyzed using linear mixed effect models including fixed effects for time, treatment, and a time-by-treatment interaction in addition to a random intercept to account for within-subject correlation. Hierarchical linear models are a robust method for analyzing ordinal outcomes using a Likert scale [47]. Secondary outcomes collected only at week 12 (i.e. pain management inventory) were analyzed using linear regression. Generalized estimating equations were used to analyze missed work and reduced activity days. The Binomial family was used to analyze the proportion of subjects with any missed work or reduced activity and the Poisson family was used to analyze the number of missed or reduced days. All models included the baseline outcome as a covariate except for analyses of improvement and satisfaction where baseline measures are not applicable.

### Primary outcome measure analysis

The primary outcomes were short-term (4 to 12 week) and long-term (4 to 52 weeks) group differences in pain intensity derived from the linear mixed effect model. We used Fisher’s protected least significant difference approach to control for multiplicity [48]. An area under the curve minus baseline summary measure was used as the omnibus test to determine if the long-term pain profile (including 4, 12, 26, and 52 weeks) was different between groups [49, 50]. The omnibus test needed to be significant (*p*-value ≤ 0.05) for group differences in the short term (weeks 4 through 12) to be determined. Clinical and demographic variables were included as covariates if they were at least moderately correlated (|r|≥ 0.5) with change in outcomes [51]. Linear mixed effect model analyses provide unbiased estimates when data are missing at random [52]. The pattern and reasons for missing data were assessed to determine whether sensitivity analyses to address data missing not at random were required. In addition, a sensitivity analysis including patient expectations as a covariate was conducted to assess impact on study results.

Secondary analyses of the primary outcome measure included group differences at weeks 4, 12, 26, and 52. Additionally, responder analyses for no pain reduction, or pain reductions of 30% (minimal improvement), 50% (moderate improvement), 75%, and 100% (substantial improvement) were performed at weeks 12, 26, and 52 [53]. Differences in proportions of responders between groups were calculated and 95% confidence intervals were analyzed using the Wilson method for risk differences [54]. Cumulative responder analysis graphs were created to display the proportion of responders for all possible levels of pain reduction [55]. Differences in cumulative response curves were assessed by determining the area under the response curve using the trapezoidal rule and 95% confidence intervals were calculated using bias-corrected bootstrapping with 5000 iterations [56].

### Secondary outcome measure analysis

Analyses included group differences at the relevant individual time points for all measures, in addition to short-term (including 4 and 12 week outcomes) and long-term (including all time points) profiles when possible.

## Results

### Baseline characteristics

**Fig. 1:**
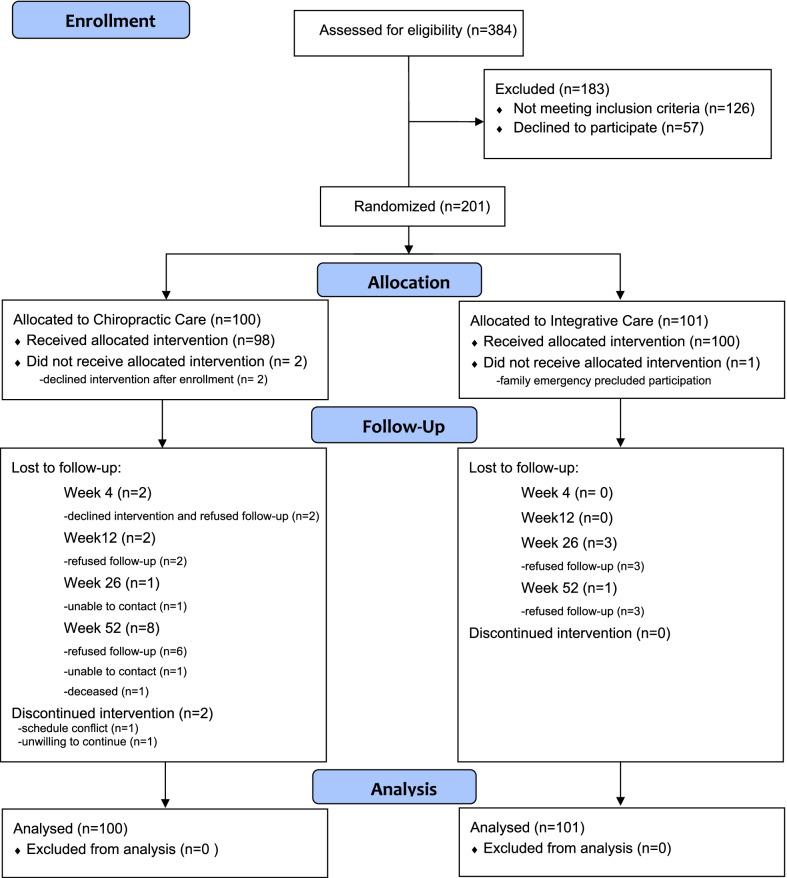
CONSORT participant flow

A summary of study participants is provided in Fig. 1. A total of 384 participants were assessed for eligibility and 201 were enrolled. Demographic and baseline clinical characteristics are provided in Table 2. On average, participants were just over 50 years of age and had pain that was chronic in nature (8 to 9 years); low back pain intensity was moderate (~ 5 on a 0–10 scale) as was disability level (~ 40 percent, 0–100). The two groups were comparable at baseline in terms of demographic and clinical characteristics. Pain intensity was the only baseline clinical or demographic characteristic that was moderately correlated with changes in pain intensity and was included as a covariate in the primary analysis. Expectation of improvement was very weakly correlated with change in pain intensity (r = − 0.13 or weaker).

**Table 2:** Baseline demographics and clinical characteristics (mean (SD) unless otherwise noted)

Parameter	Treatment group	
	Chiropractic Care	Integrated Care
n	100	101
Age	52.3 (12.4)	52.6 (12.5)
Female, n (%)	60 (60.0%)	69 (68.3%)
Non-white race, n (%)	5 (5.1%)	1 (1.0%)
Hispanic, n (%)	3 (3.0%)	5 (5.0%)
College degree, n (%)	47 (47.5%)	52 (51.5%)
Household income < $35,000, n (%)	18 (18.3%)	16 (19.8%)
Employed, n (%)	71 (71.0%)	66 (66.0%)
BMI	29.5 (5.8)	27.5 (5.2)
Duration [years]	9.2 (10.1)	8.3 (9.9)
Median [25th to 75th percentiles]	5.0 [2.0 to 15.0]	4.0 [2.0 to 11.0]
Chronic (current episode ≥ 12 weeks), n (%)	97 (97.0%)	98 (97.0%)
Radiation to lower extremity, n (%)	18 (18.0)	21 (20.8)
Back pain associated with trauma		
Auto accident, n (%)	5 (5.0%)	4 (4.0%)
Work or leisure time accident, n (%)	21 (21.0%)	21 (20.8%)
Age at first episode of back pain	35.7 (15.0)	35.7 (15.0)
Prior treatment, n (%)	88 (88.0%)	90 (89.1%)
Depression, n (%)	12 (12.0%)	11 (10.9%)
Other pain, n (%)	88 (88.0%)	89 (88.1%)
Tobacco use, n (%)	9 (9.0%)	10 (9.9%)
Low back pain intensity [0 to 10]	5.4 (1.5)	5.1 (1.6)
Low back disability (Roland Morris) [0 to 100]^†^	40.9 (21.3)	38.1 (19.2)
Quality of life (EuroQol) [− 0.109 to 1]*	0.76 (0.10)	0.79 (0.06)
Medication use (days/week)	2.8 (2.4)	2.9 (2.4)
Preferred intervention, n (%)		
None	19 (19.4%)	17 (17.2%)
Acupuncture and oriental medicine	25 (25.5%)	23 (23.2%)
Chiropractic	19 (19.4%)	17 (17.2%)
Cognitive behavioral therapy	0 (0.0%)	0 (0.0%)
Exercise therapy	11 (11.2%)	11 (11.1%)
Massage	22 (22.4%)	31 (31.3%)
Medication	1 (1.0%)	0 (0.0%)
Self-care education	1 (1.0%)	0 (0.0%)
Expectation of improvement at the end of treatment (1–5) ^	1.80 (0.53)	1.77 (0.46)

^†^Lower scores indicate lower disability;

*Higher scores indicate higher quality of life;

^Lower scores indicate higher expectations

### Treatment frequency and adherence

Overall, 96% of study participants attended treatment visits as recommended; 93% for the CC group and 98% in the IC group. The mean number of visits in the CC group was 18.1 and for the IC group, was 23.8. Participants in the IC group received the following types of care: exercise therapy (ET, n = 96); self-care education (SCE, n = 59); traditional Chinese medicine (TCM, n = 51); massage therapy (MT, n = 37); chiropractic care (CC, n = 19); cognitive behavioral therapy (CBT, n = 35) and medication (MED, n = 5). The most frequent combinations were: TCM/SCE/ET (n = 22); ET, SCE, MT (n = 10); and ET, SCE, MT, CBT (n = 10). All participants received at least two types of treatment, and 27 received at least four. One participant received all of the treatments.

During the 12-week intervention, 4 participants reported visits to other health care providers for their LBP: 3 from the CC group and 1 from the IC group. Between weeks 12 and 52, a total of 83 individuals sought additional health care: 46 in CC and 37 in IC.

### Effectiveness assessments

#### Primary analysis of primary outcome measure

**Fig. 2:**
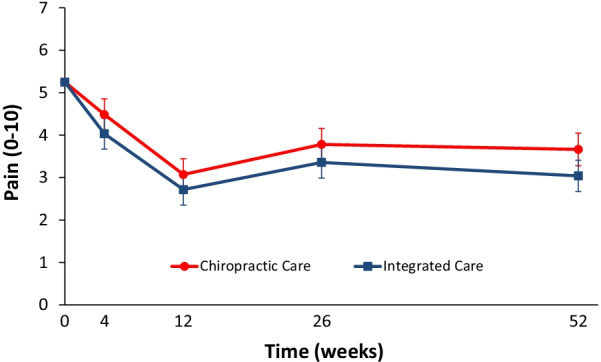
Mean Pain Reduction with 95% Confidence Intervals

The longitudinal omnibus test for pain showed IC to be significantly superior to CC over the 1-year period (*P* = 0.02). The long-term profile for pain intensity (0–10) which included data from weeks 4 through 52, showed a significant advantage of 0.5 for IC over CC (95% CI 0.1 to 0.9; *P* = 0.02). The short-term profile (weeks 4 to 12) favored IC by 0.4, but was not statistically significant (95% CI − 0.02 to 0.9; *P* = 0.06). Primary results are shown in Table 3 and Fig. 2.

**Table 3:** Primary outcome measure—Low back pain intensity

	Treatment group		Group difference	*P* value*
	Chiropractic care	Integrated care	Chiropractic care minus integrated care	
Low back pain intensity [0 to 10; 0 = no LBP, 10 = the worst LBP possible]				
Mean at baseline (SD)	5.4 (1.5)	5.1 (1.6)		
Mean at week 4 (95%CI)	4.5 (4.1 to 4.9)	4.0 (3.7 to 4.4)	0.42 (−0.02 to 0.86)	0.07
Mean at week 12 (95%CI)	3.1 (2.7 to 3.4)	2.7 (2.4 to 3.1)	0.37 (−0.12 to 0.85)	0.14
Short term response summary (area under the curve minus baseline through week 12)			0.41 (−0.02 to 0.85)	0.06
Mean at week 26 (95%CI)	3.8 (3.4 to 4.2)	3.4 (3 to 3.7)	0.45 (−0.13 to 1.04)	0.13
Mean at week 52 (95%CI)	3.7 (3.3 to 4)	3.0 (2.7 to 3.4)	0.62 (0.04 to 1.21)	0.04
Long term response summary (area under the curve minus baseline through week 52)			0.46 (0.07 to 0.86)	0.02

Mean values adjusted for baseline

*Long term response summary serves as the omnibus test *p*-value. If *p* > .05, *p*-values for short term response summary and individual time points are not computed

#### Secondary analysis of primary outcome measure

**Fig. 3:**
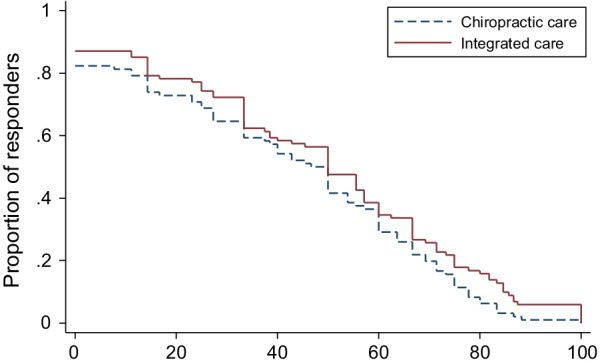
Percent reduction of LBP intensity at week 12

**Fig. 4:**
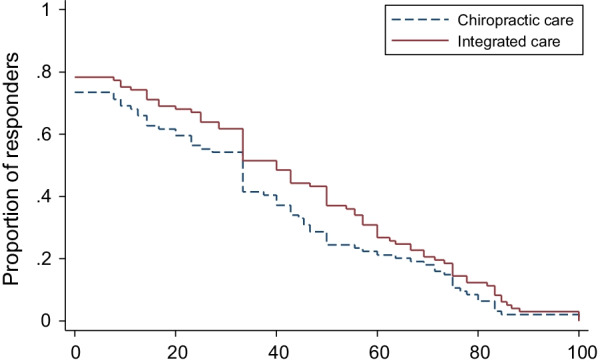
Percent reduction of LBP intensity at week 26

**Fig. 5:**
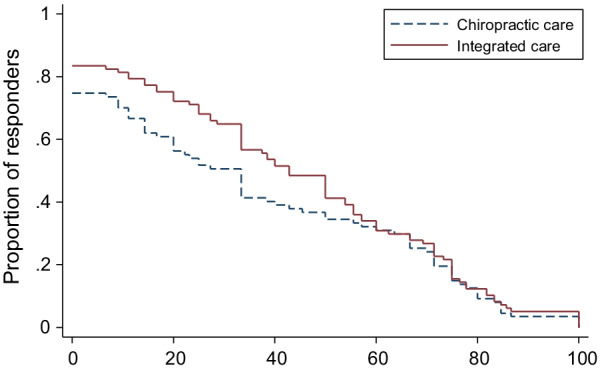
Percent reduction of LBP intensity at week 52

Group differences for pain intensity at individual time points favored IC and ranged from 0.4 (weeks 4 and 12) to 0.6 (week 52), with the only significant finding occurring at week 52 (95% CI 0.04 to 1.2; *P* = 0.04). On average, the difference in proportions for reduction of LBP intensity across all possible thresholds for improvement favored IC by approximately 6% at 12 weeks (95% CI − 3 to 13%), 7% at 26 weeks (95% CI − 2 to 15%), and 7% at 52 weeks (95% CI − 2 to 16%). Detailed results from the responder analyses are provided in Table 4 and Figs. 3, 4, 5.

**Table 4:** Responder analysis

% Pain reduction	Treatment groups		Group differences
	Chiropractic Care (%)	Integrated care (%)	Chiropractic care minus integrated care
Week 12*			
No reduction or increase	17.7	12.9	4.8 (− 5.3 to 15.1)
≥ 30%	64.6	72.3	−7.7 (−20.3 to 5.2)
≥ 50%	50.0	56.4	−6.4 (−19.9 to 7.4)
≥ 75%	15.6	21.8	−6.2 (−16.9 to 4.9)
100%	1.0	5.9	−4.9 (−11.4 to 0.7)
Week 26^^^			
No reduction or increase	26.6	21.6	4.9 (−7.2 to 16.9)
≥ 30%	54.3	61.9	−7.6 (−21.1 to 6.3)
≥ 50%	28.7	43.3	−14.6 (−27.4 to −1.0)
≥ 75%	14.9	18.6	−3.7 (−14.3 to 7.1)
100%	2.1	3.1	−1.0 (−6.8 to 4.7)
Week 52^†^			
No reduction or increase	25.3	16.5	8.8 (−2.9 to 20.5)
≥ 30%	50.6	64.9	−14.4 (−27.9 to −0.1)
≥ 50%	36.8	48.5	−11.7 (−25.2 to 2.6)
≥ 75%	19.5	21.6	−2.1 (−13.6 to 9.7)
100%	3.4	5.2	−1.7 (−8.4 to 5.2)

Proportion of participants with at least 30, 50, 75, or 100% reduction in pain intensity

*Analysis included 96 participants in Chiropractic care group and 101 in Integrated care group;

^^^Analysis included 94 participants in Chiropractic care group and 97 in Integrated care group;

^†^Analysis included 87 participants in Chiropractic care group and 97 in Integrated care group;

#### Analysis of secondary outcome measures

**Fig. 6:**
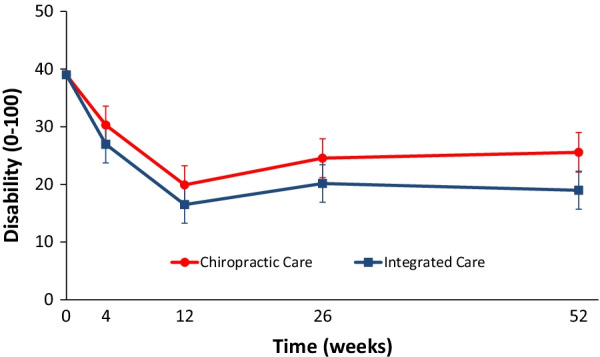
Mean disability reduction with 95% confidence intervals

**Fig. 7:**
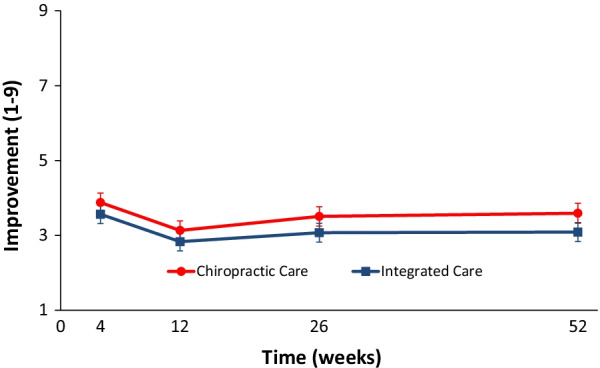
Mean improvement with 95% confidence intervals

Long term longitudinal profiles significantly favored IC over CC for disability (Fig. 6), improvement (Fig. 7), satisfaction, and low back symptom frequency (Table 5). Medication use, quality of life, leg symptom frequency, fear avoidance beliefs, and self-efficacy did not significantly differ between groups over the 1-year period. Cross-sectional group differences for secondary outcomes mainly favored IC over CC, but most differences were not significant. The exceptions were improvement, satisfaction with care, and frequency of low back symptoms with IC consistently demonstrating a significant advantage over CC. IC also demonstrated a significant advantage over CC for passive coping strategies at week 12 in addition to disability and self-efficacy at week 52.

**Table 5:** Participant-reported secondary outcome measures

	Treatment group		Group difference	*P* value*
	Chiropractic care	Integrated care	Chiropractic care minus integrated care	
Low back disability (Roland Morris) [0 to 100; lower scores indicate less disability]				
Mean at baseline (SD)	40.9 (21.3)	38.1 (19.2)		
Mean at week 4 (95% CI)	30.3 (27 to 33.6)	27.0 (23.7 to 30.2)	3.07 (− 0.95 to 7.08)	0.14
Mean at week 12 (95% CI)	19.9 (16.6 to 23.2)	16.5 (13.3 to 19.7)	3.66 (− 0.65 to 7.97)	0.10
Short term response summary (area under the curve minus baseline through week 12)			3.36 (− 0.65 to 7.38)	0.10
Mean at week 26 (95% CI)	24.6 (21.2 to 27.9)	20.2 (16.9 to 23.4)	4.05 (− 0.97 to 9.07)	0.12
Mean at week 52 (95% CI)	25.6 (22.1 to 29)	19.0 (15.7 to 22.3)	6.54 (1.18 to 11.90)	0.02
Long term response summary (area under the curve minus baseline through week 52)			4.62 (0.88 to 8.36)	0.02
Improvement [1 to 9; 1 = 100% Improvement, 9 = 100% Worse]				
Mean at week 4 (95% CI)	3.9 (3.6 to 4.1)	3.6 (3.3 to 3.8)	0.31 (0.03, 0.60)	0.03
Mean at week 12 (95% CI)	3.1 (2.9 to 3.4)	2.8 (2.6 to 3.1)	0.29 (− 0.04, 0.63)	0.09
Short term response summary (Area under the curve through week 12)			0.31 (0.01 to 0.61)	0.04
Mean at week 26 (95% CI)	3.5 (3.2 to 3.8)	3.1 (2.8 to 3.3)	0.38 (0.08, 0.68)	0.01
Mean at week 52 (95% CI)	3.6 (3.3 to 3.9)	3.1 (2.8 to 3.3)	0.37 (0.08, 0.65)	0.01
Long term response summary (area under the curve through week 52)			0.41 (0.14 to 0.68)	< 0.01
Medication use [days/week]				
Mean at baseline (SD)	2.8 (2.4)	2.9 (2.4)		
Mean at week 4 (95% CI)	2.3 (1.9 to 2.7)	2.6 (2.2 to 3)	− 0.27 (− 0.77 to 0.24)	
Mean at week 12 (95% CI)	1.8 (1.4 to 2.2)	1.8 (1.4 to 2.2)	− 0.05 (− 0.63 to 0.52)	
Short term response summary (area under the curve minus baseline through week 12)			− 0.21 (− 0.71 to 0.30)	
Mean at week 26 (95% CI)	1.8 (1.4 to 2.2)	2.2 (1.8 to 2.6)	− 0.44 (− 1.06 to 0.19)	
Mean at week 52 (95% CI)	2.3 (1.9 to 2.8)	1.9 (1.5 to 2.4)	0.50 (− 0.17 to 1.17)	
Long term response summary (area under the curve minus baseline through week 52)			− 0.11 (− 0.58 to 0.36)	0.65
Quality of life (EuroQol) [− 0.109 to 1; higher scores indicate better quality of life]				
Mean at baseline (SD)	0.76 (0.10)	0.79 (0.06)		
Mean at week 4 (95% CI)	0.80 (0.78 to 0.82)	0.81 (0.79 to 0.83)	− 0.01 (− 0.04 to 0.02)	
Mean at week 12 (95% CI)	0.83 (0.81 to 0.85)	0.85 (0.83 to 0.87)	− 0.02 (− 0.05 to 0.01)	
Short term response summary (area under the curve minus baseline through week 12)			− 0.01 (− 0.04 to 0.01)	
Mean at week 26 (95% CI)	0.81 (0.79 to 0.83)	0.82 (0.80 to 0.84)	− 0.02 (− 0.04 to 0.01)	
Mean at week 52 (95% CI)	0.82 (0.79 to 0.84)	0.84 (0.82 to 0.86)	− 0.02 (− 0.05 to 0.004)	
Long term response summary (area under the curve minus baseline through week 52)			− 0.02 (− 0.04 to 0.003)	0.09
Satisfaction with care [1 to 7; 1 = Completely Satisfied, 7 = Completely Dissatisfied]				
Mean at week 4 (95% CI)	2.1 (1.9 to 2.3)	1.8 (1.6 to 2)	0.34 (0.12, 0.57)	< 0.01
Mean at week 12 (95% CI)	2 (1.8 to 2.2)	1.7 (1.5 to 1.9)	0.33 (0.09, 0.56)	< 0.01
Short term response summary (area under the curve minus baseline through week 12)			0.34 (0.11 to 0.56)	< 0.01
Mean at week 26 (95% CI)	2.3 (2.1 to 2.5)	1.9 (1.7 to 2.1)	0.38 (0.08, 0.68)	0.01
Mean at week 52 (95% CI)	2.2 (2 to 2.4)	1.8 (1.6 to 2)	0.37 (0.08, 0.65)	0.01
Long term response summary (area under the curve through week 52)			0.38 (0.16 to 0.59)	< 0.01
Low back symptom frequency [0 to 5; 0 = none of the time, 5 = all of the time]				
Mean at baseline (SD)				
Mean at week 4 (95% CI)	3.11 (2.9 to 3.32)	2.83 (2.62 to 3.04)	0.28 (− 0.01 to 0.58)	0.06
Mean at week 12 (95% CI)	2.38 (2.17 to 2.59)	2.02 (1.81 to 2.23)	0.36 (0.06 to 0.66)	0.02
Short term response summary (Area under the curve minus baseline through week 12)			0.31 (0.07 to 0.56)	0.01
Mean at week 26 (95% CI)	2.68 (2.47 to 2.9)	2.33 (2.12 to 2.54)	0.35 (0.05 to 0.66)	0.02
Mean at week 52 (95% CI)	2.41 (2.19 to 2.64)	1.95 (1.74 to 2.16)	0.46 (0.16 to 0.77)	0.003
Long term response summary (area under the curve through week 52)			0.38 (0.15 to 0.60)	0.001
Leg symptom frequency [0 to 5; 0 = none of the time, 5 = all of the time]				
Mean at baseline (SD)				
Mean at week 4 (95% CI)	1.1 (0.92 to 1.28)	1.03 (0.85 to 1.21)	0.07 (− 0.19 to 0.32)	
Mean at week 12 (95% CI)	0.77 (0.58 to 0.95)	0.66 (0.49 to 0.84)	0.1 (− 0.15 to 0.36)	
Short term response summary (Area under the curve minus baseline through week 12)			0.08 (− 0.13 to 0.29)	
Mean at week 26 (95% CI)	0.94 (0.75 to 1.12)	0.87 (0.69 to 1.05)	0.07 (− 0.2 to 0.33)	
Mean at week 52 (95% CI)	1.04 (0.85 to 1.24)	0.79 (0.6 to 0.97)	0.26 (− 0.01 to 0.52)	
Long term response summary (Area under the curve through week 52)			0.12 (− 0.06 to 0.31)	0.19
Any missed work days due to back problem in past month [%]				
Percentage at baseline (n)	36.3 (36)	36.6 (37)		
Percentage at week 4 (95% CI)	23.5 (16.4 to 30.5)	24.2 (17.4 to 31.0)	− 0.7 (− 10.2 to 8.7)	
Percentage at week 12 (95% CI)	17.5 (11.0 to 24.1)	13.0 (8.2 to 17.8)	4.5 (− 3.1 to 12.1)	
Percentage at week 26 (95% CI)	15.2 (9.4 to 21.1)	16.6 (10.6 to 22.6)	− 1.3 (− 9.1 to 6.5)	
Percentage at week 52 (95% CI)	17.3 (10.5 to 24.1)	16.1 (9.9 to 22.3)	1.2 (− 7.6 to 10.0)	
Number of missed work days due to back problem in past month [0–31 days]				
Mean at baseline (SD)	6.0 (4.9)	6.6 (6.8)		
Mean at week 4 (95% CI)	6.09 (3.56 to 8.62)	4.49 (3.13 to 5.85)	1.60 (− 1.29 to 4.49)	
Mean at week 12 (95% CI)	2.18 (1.35 to 3.01)	3.84 (1.55 to 6.12)	− 1.65 (− 4.12 to 0.81)	
Mean at week 26 (95% CI)	6.23 (1.33 to 11.14)	5.38 (0.54 to 10.21)	0.86 (− 6.07 to 7.78)	
Mean at week 52 (95% CI)	3.16 (1.98 to 4.33)	6.11 (0.20 to 12.02)	− 2.96 (− 9.03 to 3.11)	
Any reduced activity days due to back problem in past month [%]				
Percentage at baseline (n)	74.0 (74)	72.2 (73)		
Percentage at week 4 (95% CI)	54.4 (45.7 to 63.1)	64.8 (56.5 to 73.1)	− 10.4 (− 22.4 to 1.6)	
Percentage at week 12 (95% CI)	41.3 (31.4 to 51.2)	41.8 (32.9 to 50.8)	− 0.6 (− 13.9 to 12.8)	
Percentage at week 26 (95% CI)	43.7 (34.7 to 52.7)	37.1 (30.0 to 44.2)	6.6 (− 4.4 to 17.6)	
Percentage at week 52 (95% CI)	41.5 (32.6 to 50.4)	34.7 (26.9 to 42.5)	6.8 (− 4.5 to 18.2)	
Number of reduced activity days due to back problem in past month [0–31 days]				
Mean at baseline (SD)	9.0 (8.2)	8.4 (8.0)		
Mean at week 4 (95% CI)	6.05 (5.02 to 7.08)	5.07 (4.19 to 5.95)	0.98 (− 0.26 to 3.01)	
Mean at week 12 (95% CI)	5.04 (3.10 to 6.99)	4.22 (3.10 to 5.35)	0.82 (− 1.37 to 3.01)	
Mean at week 26 (95% CI)	4.58 (3.56 to 5.60)	4.53 (3.55 to 5.51)	0.05 (− 1.24 to 1.33)	
Mean at week 52 (95% CI)	4.84 (3.36 to 6.33)	4.53 (3.54 to 5.52)	0.31 (− 1.34 to 1.96)	
Fear avoidance beliefs—work subscale [0–42; higher scores indicate greater fear avoidance beliefs]				
Mean at baseline (SD)	9.13 (8.94)	9.69 (8.47)		
Mean at week 4 (95% CI)	8.30 (7.15 to 9.45)	7.53 (6.4 to 8.66)	0.77 (− 0.84 to 2.38)	
Mean at week 12 (95% CI)	7.10 (5.94 to 8.26)	5.94 (4.82 to 7.06)	1.16 (− 0.45 to 2.77)	
Short term response summary (area under the curve minus baseline through week 12)			0.93 (− 0.46 to 2.31)	
Mean at week 26 (95% CI)	6.80 (5.62 to 7.98)	5.65 (4.51 to 6.78)	1.15 (− 0.48 to 2.79)	
Mean at week 52 (95% CI)	6.60 (5.39 to 7.81)	6.49 (5.34 to 7.64)	0.11 (− 1.56 to 1.78)	
Long term response summary (area under the curve through week 52)			0.84 (− 0.44 to 2.11)	0.20
Fear avoidance beliefs—physical activity subscale [0 to 24; higher scores indicate greater fear avoidance beliefs]				
Mean at baseline (SD)	11.66 (5.42)	11.63 (5.32)		
Mean at week 4 (95% CI)	9.03 (8.04 to 10.01)	9.3 (8.33 to 10.27)	− 0.28 (− 1.66 to 1.11)	
Mean at week 12 (95% CI)	7.54 (6.55 to 8.54)	7.36 (6.39 to 8.33)	0.18 (− 1.21 to 1.57)	
Short term response summary (Area under the curve minus baseline through week 12)			− 0.09 (− 1.31 to 1.12)	
Mean at week 26 (95% CI)	7.71 (6.7 to 8.72)	6.52 (5.53 to 7.5)	1.20 (− 0.21 to 2.6)	
Mean at week 52 (95% CI)	7.61 (6.57 to 8.64)	7.22 (6.23 to 8.21)	0.39 (− 1.04 to 1.82)	
Long term response summary (area under the curve through week 52)			0.59 (− 0.55 to 1.72)	0.31
Self-efficacy [0 to 60; higher scores indicate greater self-efficacy]				
Mean at baseline (SD)	48.0 (10.0)	49.2 (8.7)		
Mean at week 4 (95% CI)	52.2 (50.9 to 53.6)	52 (50.7 to 53.4)	0.18 (− 1.70 to 2.07)	
Mean at week 12 (95% CI)	54.4 (53 to 55.7)	54.7 (53.4 to 56)	− 0.33 (− 2.22 to 1.57)	
Short term response summary (area under the curve minus baseline through week 12)			− 0.02 (− 1.67 to 1.63)	
Mean at week 26 (95% CI)	52.5 (51.2 to 53.9)	53.2 (51.8 to 54.5)	− 0.63 (− 2.55 to 1.29)	
Mean at week 52 (95% CI)	51.8 (50.4 to 53.2)	53.8 (52.5 to 55.2)	− 1.99 (− 3.94 to − 0.04)	
Long term response summary (area under the curve through week 52)			− 0.82 (− 2.35 to 0.71)	0.29
Active pain coping strategies (pain management inventory) [5 to 25; higher scores indicate more frequent use]				
Mean at baseline (SD)	17.3 (3.4)	17.1 (3.6)		
Mean at week 12 (95% CI)	18.2 (17.6 to 18.9)	19.0 (18.4 to 19.7)	− 0.8 (− 1.7 to 0.1)	0.07
Passive pain coping strategies (Pain Management Inventory) [6 to 30; higher scores indicate more frequent use]				
Mean at baseline (SD)	13.8 (4.5)	13.9 (3.9)		
Mean at week 12 (95% CI)	12.6 (12.0 to 13.2)	11.7 (11.1 to 12.3)	0.9 (0.1 to 1.7)	0.036
Kinesiophobia [17 to 68] (11 questions; 1 = Strongly Disagree—4 = Strongly Agree)				
Mean at baseline (SD)	35.2 (6.3)	33.2 (5.8)		
Mean at week 12 (95% CI)	29.7 (28.6 to 30.8)	28.7 (27.6 to 29.8)	1.0 (2.6 to − 0.6)	0.20

Mean values adjusted for baseline except for improvement and satisfaction

*Long term response summary serves as the omnibus test *p*-value. If *p* > .05, *p*-values for short term response and individual time points are not computed

#### Missing data and sensitivity analyses

Among the 201 participants, 182 (91%) provided data on back pain intensity at every time point, and 166 (83%) provided the secondary outcomes at every time point. A total of 14 participants in the CC group and 5 in the IC group did not provide primary outcome data at all time points and the pattern of missingness seemed to be nonrandom. Participants with missing data at any time point reported higher pain intensity (when data was available) than participants with no missing data and this pattern was similar between groups. Sensitivity analyses for data missing not at random were performed using pattern mixture methods [57]. Missing pain intensity outcomes were imputed separately for each treatment group using multiple imputation (Procedure MI in STATA). Five imputed data sets were created using a multivariate normal model including baseline covariates associated with missing data. The sensitivity analysis for data missing not at random assumed the imputed pain intensity outcomes were worse by 50%. The estimated group differences from the missing data sensitivity analyses were similar in magnitude and in the same direction as the primary analysis, and all statistically significant between-group differences remained. The sensitivity analysis adjusting for expectations led to very small increases of group differences ranging from 0.3 to 1.1 percentage points across all time points.

#### Adverse events

There were five serious adverse events (SAEs) that occurred during the course of the trial (CC = 4, IC = 1); all were classified as unrelated to study interventions. Four SAEs were reported by patients assigned to CC in which three were hospitalized (pneumonia, surgical intervention for fractured foot, and syncope), and one diagnosed with a brain tumor. One patient assigned to IC was hospitalized for nephrolithiasis evaluation and management.

Minor self-limiting adverse events during the 12 weeks of intervention were reported with about equal frequency in both groups (Table 6). The most commonly reported participants were unusual or increased soreness (51–54%) and a different type of pain (31–34%).

**Table 6:** Adverse events during the 12-week treatment*

	Treatment group				Group difference
	Chiropractic care		Integrated care		Chiropractic care minus Integrated care (95% CI)
	n^†^ (%)	Median bothersomeness ^	n^†^ (%)	Median bothersomeness ^	
Different type of pain	33 (33.7%)	6	35 (34.7%)	5	− 1.0 (− 13.9 to 12.0)
Increased back pain intensity	20 (20.4%)	5	23 (22.8%)	5	− 2.4 (− 13.7 to 9.1)
New or increased leg pain, numbness, or weakness	16 (16.3%)	4	13 (12.9%)	2	3.5 (− 6.5 to 13.5)
Unusual or increased soreness	41 (41.8%)	3	47 (46.5%)	3	− 4.7 (− 18.1 to 9.0)
Skin irritation	4 (4.1%)	7	5 (5.0%)	2	− 0.9 (− 7.5 to 5.7)
More fatigue than usual	14 (14.3%)	5	13 (12.9%)	4	1.4 (− 8.3 to 11.2)
Dizziness or lightheadedness	11 (11.2%)	4	12 (11.9%)	3	− 0.7 (− 9.8 to 8.6)
Upset stomach, nausea, or vomiting	6 (6.1%)	4	5 (5.0%)	2	1.2 (− 5.8 to 8.3)
Changes in bowel or bladder habits	4 (4.1%)	1	13 (12.9%)	0.5	− 8.8 (− 17.1 to − 0.9)

*Analysis included 98 participants in Chiropractic care group and 101 in Integrated care group;

^†^Participants reporting at least one event during treatment, participants could report more than one event

^Bothersomeness on 0–10 scale; bothersomeness was averaged for participants with more than one of the same event during the 12 weeks of treatment

## Discussion

### Summary of findings

While there have long been calls to address LBP from a more comprehensive biopsychosocial perspective, there is still little research to date that has done so in a rigorous fashion. This study examines one approach for remedying this gap by comparing an integrative care (IC) intervention applying a multidisciplinary team-based approach to monodisciplinary chiropractic care. Overall, analyses demonstrated a consistent trend in favor of the IC group.

### Clinical importance

Discussions of clinical importance for group differences requires consideration of the broader context regarding the condition being studied, what treatments are available, and the overall risk–benefit ratio of available options, which goes beyond criteria for establishing a clinically important change at the individual patient level [58]. Several factors should be considered when assessing the clinical importance of study results including magnitude of group differences in primary and secondary outcomes, proportion of responders, consistency of findings across outcomes, durability of effects, adverse events, treatment adherence, and costs [58]. While there was an advantage for the IC group in terms of the primary outcome, pain intensity over 1 year, the magnitude of the group difference fell below the threshold of a moderate effect size that was used to power the study. Further, despite the IC group consistently reporting greater percentage reductions of pain intensity than the CC group, they were generally small (< 10%) with the exception of the 50% threshold at Week 26 and the 30% threshold at Week 52. There was a significant advantage over the long term for IC in terms of some secondary measures (disability, improvement, satisfaction and low back symptom frequency), but not for others (medication use, quality of life, leg symptom frequency, fear avoidance beliefs, self-efficacy, active pain coping, and kinesiophobia). Importantly, no serious adverse events resulted from either of the interventions, and less serious events were approximately equal in both groups. Functional change in objective measures of torso strength and endurance, as well as lumbar dynamic motion characteristics, may provide further clinical implications and it is our intent to analyze and report these outcomes in future manuscripts. Similarly, qualitative analysis of interviews collected pre and post study invention will give insight to the patients’ perspective. Finally, while a formal cost-effectiveness analysis has yet to be performed, the IC group did require substantially more resources to implement, and patients in that group had more visits raising the question of cost–benefit. When considered together, these factors suggest that while the IC group tended to do better in the long term on some important outcomes, the overall clinical importance of these findings remains debatable for chronic LBP populations similar to the one studied.

### Comparison with other studies

The most recent Cochrane systematic review by Kamper et al. [20] examining multidisciplinary biopsychosocial rehabilitation programs for chronic LBP found a modest advantage compared to usual care or physical treatments for reducing pain and disability and increasing the likelihood of return to work. We have identified 6 additional trials published after the Cochrane review which had similar magnitudes of improvement in pain intensity and disability relative to physical treatments, generally confirming our findings [59–65]. Two low risk of bias trials conducted by Monticone et al. are notable exceptions [59, 60]. These trials reported much larger treatment effects for a multidisciplinary program incorporating CBT with manual treatment and task-oriented exercise compared to manual therapy and exercise, with average reductions in pain intensity and disability around 50–75%.

The Kamper et al. review reported on subgroup analyses assessing the impact of baseline symptom severity on the effectiveness of the multidisciplinary programs and found the results were inconclusive; however, given the modest benefits and relatively high resource and cost burden, the authors suggest these programs be reserved for the most severe cases where marked psychosocial distress is present. This approach is consistent with emerging risk-stratification and stepped care models aimed towards improving the efficiency and quality of care for musculoskeletal conditions [66].

### Strengths and limitations

This study had several strengths, including the long-term follow up and high intervention adherence and follow up data collection rates. Expectations, a potentially important contextual factor that can influence treatment outcomes [67, 68] were measured, and were found to be similar between groups and had little impact on the primary outcome. Importantly, side effects and adverse events were systematically collected and reported (see Table 6). Another strength was the development and application of a clinical care pathway that integrated the best available evidence with patient-rated outcome instruments and patient preferences to create biopsychosocial patient profiles to inform team based shared decision making [23].

As with any study there are also limitations that must be considered when interpreting the results. An important one which is common to many studies of complementary therapies, is the lack of representation of individuals from more diverse backgrounds, especially in terms of race and income. Also, despite careful attention to applying a clinical care pathway process [23], it was not validity and reliability tested, limiting its replication to other research and clinical settings, and potentially impacting the results of this trial. For example, while an important aspect of the pain pathway included assessing patients’ psychosocial needs with established patient rated outcome instruments, treatment plans were still very much oriented towards managing pain, versus the whole person with pain, which is advocated for a truly biopsychosocial approach [11]. This was evidenced by providers recommending and patients mainly choosing treatments that were focused on physical symptoms and function in the IC group [23]. Of note is that social factors were not thoroughly addressed which is a common limitation of current pain research and should be remedied in future trials [11]. Admittedly, assessing psychosocial risk is still an area in the LBP field that remains relatively underdeveloped [69, 70]. Patients in this study had relatively low baseline measures of fear-avoidance beliefs, and high self-efficacy and active pain coping which all would suggest these patients to be only modestly psychosocially impacted, and which potentially explains the lack of use of CBT in this study.

Because of the pragmatic nature of the study with intervention delivery designed to approximate how it could be implemented in practice, it is difficult to discern between the active elements of treatment and potentially important contextual and structural intervention qualities (e.g. influence of the practitioner, therapeutic relationship, number of visits, time spent, etc.). Additionally, fidelity assessments of the interventions were limited to documentation by providers and patient-self report. Finally, another limitation of the study is the time it has taken from completion of the trial to publishing of the results, which came about from several members of the investigative team changing institutions. The findings however remain very relevant especially in light of continued and pervasive use of biophysically focused mono-disciplinary treatments (e.g. medications, surgery, etc.) that still plague the LBP field, despite their limited effectiveness.

### Implication for clinical practice

Team based models of care with multiple provider types from complementary disciplines has been one way of integrating different therapies to more comprehensively address patients’ needs [11, 20]. However, these approaches can have significant challenges including inconvenience and inaccessibility due to multiple appointments with different providers along with substantial system resources needed to coordinate care across provider types, all which can contribute to disjointed and unsatisfactory care [11]. While our study was able to successfully coordinate multidisciplinary treatment plans for participants as evidenced by high adherence and satisfaction rates, achieving this required significant time and resources which is a major barrier in most clinical settings [11, 23]. Further, the findings of this trial, along with other research evidence, suggest only modest advantages in terms of clinical outcomes of multidisciplinary team based interventions [20]. Importantly, the IC group in this study had a mean of nearly 24 visits compared to 18 in the CC group, with many of the visit lengths substantially longer than a typical CC visit. This has potential cost implications to both systems and patients, posing additional hurdles to effective pain care, especially for those with challenging socioeconomic circumstances. It is likely that offering these resource intensive approaches are not going to be cost-effective. As one example, cost-effectiveness comparisons of chiropractic care have been shown to be more advantageous to more structurally intensive interventions for older neck pain patients [71].

### Implication for future research

One potential alternative to integrative multidisciplinary interventions is to train individual front-line providers to address patients’ biopsychosocial needs within the scope of their professional discipline, using multiple modalities to support guideline recommendations. Indeed, there have been shifts in both the chiropractic and physical therapy fields to take such an approach, integrating more psychosocial elements to these professional care models [72–75]. To facilitate, future attention is required to developing translational support tools to comprehensively address the full range of patients’ biopsychosocial needs in a manner that facilitates shared decision making and monitoring in an accurate, systematic and practical manner [66, 76].

Additionally, future studies should consider ‘intervention mapping’ in the trial design phase, where the mechanisms of action of each element of multimodal care packages are more carefully aligned with patient needs and perhaps a greater range of more relevant psychosocial outcomes. This can facilitate results interpretations, as well as reporting and replication of interventions, and ultimately broader dissemination to those who could most benefit [77, 78]. Greater attention should also be given to fidelity assessment (i.e. video recordings) to ensure interventions are being delivered as intended. Finally, given the resource intensive nature of the IC group, cost-effectiveness analyses comparing CC to IC from societal and healthcare perspectives are warranted; these are forthcoming in a future publication.

## Conclusion

Low back pain patients who received integrative care by a multidisciplinary integrative care team tended to have better outcomes than those who received chiropractic care. However, given the relatively small magnitude of between group differences and the extensive resources required to successfully manage and implement, the team based integrative care might not be worthwhile. More efficient models for addressing biopyschosocial care for low back pain should be explored with greater emphasis on addressing the full spectrum of related psychosocial mechanisms and ensuring equitable access for all.

## Data Availability

The data analyzed during the current study are available from the corresponding author on reasonable request.
